# Brillouin-scattering-induced transparency and non-reciprocal light storage

**DOI:** 10.1038/ncomms7193

**Published:** 2015-02-04

**Authors:** Chun-Hua Dong, Zhen Shen, Chang-Ling Zou, Yan-Lei Zhang, Wei Fu, Guang-Can Guo

**Affiliations:** 1 Key Laboratory of Quantum Information, University of Science and Technology of China, Hefei 230026, P. R. China; 2 Synergetic Innovation Center of Quantum Information and Quantum Physics, University of Science and Technology of China, Hefei 230026, P. R. China

## Abstract

Stimulated Brillouin scattering is a fundamental interaction between light and travelling acoustic waves and arises primarily from electrostriction and photoelastic effects, with an interaction strength several orders of magnitude greater than that of other relevant non-linear optical processes. Here we report an experimental demonstration of Brillouin-scattering-induced transparency in a high-quality whispering-gallery-mode optical microresonantor. The triply resonant Stimulated Brillouin scattering process underlying the Brillouin-scattering-induced transparency greatly enhances the light–acoustic interaction, enabling the storage of light as a coherent, circulating acoustic wave with a lifetime up to 10 μs. Furthermore, because of the phase-matching requirement, a circulating acoustic wave can only couple to light with a given propagation direction, leading to non-reciprocal light storage and retrieval. These unique features establish a new avenue towards integrated all-optical switching with low-power consumption, optical isolators and circulators.

## Abstract (web summary)

Stimulated Brillouin scattering is a non-linear interaction that allows light to be stored as coherent acoustic waves. Here, the authors report on Brillouin scattering-induced transparency in an optical microresonator whose high quality allows for long-lifetime non-reciprocal light storage.

## Graphical abstract

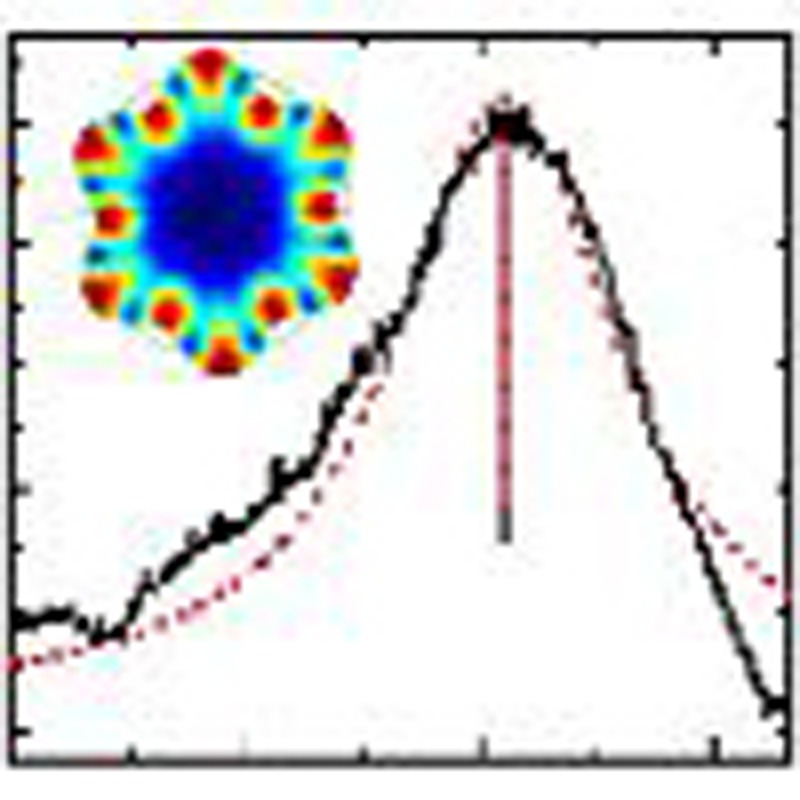

Stimulated Brillouin Scattering (SBS) in fibres and waveguides has attracted strong interest in a variety of photonic applications during past decades^1^,^2^, such as light storage^3^, slow light^4^,^5^, lasers^6^ and optical isolators^7^,^8^. SBS can be incorporated into photonic integrated circuits^9^, where the tight confinement of the optical fields greatly enhances the SBS interaction^10^. Giant enhancement of SBS in the subwavelength scales due to radiation pressures or boundary-induced non-linearities has been demonstrated recently^10^,^11^. SBS has also been realized in optical microresonators, such as silica microspheres^12^ and disks^13^, crystalline cylinders^14^. In these whispering gallery resonators, optical and acoustic waves circulate along the equatorial surface, forming optical and mechanical whispering-gallery modes (WGMs) with ultrahigh-quality (*Q*) factor. The SBS process can become triply resonant when the control field, the Stokes or anti-Stokes field and the acoustic wave are all resonant with the relevant optical and mechanical modes. The triply resonant SBS process, along with the ultrahigh *Q*-factors and the small mode volume, provides new opportunities for exploring coherent light–acoustic interactions. Over the past few years, low-threshold Brillouin lasers^14^, Brillouin optomechanics^15^,^16^ and Brillouin cooling^17^,^18^ have been reported in such triply resonant WGMs.

Here we report the experimental demonstration of Brillouin-scattering-induced transparency (BSIT) and non-reciprocal light storage in a silica microsphere resonator. In contrast to the optomechanically induced transparency that has been observed in a variety of optomechanical systems^19^,^20^, two optical modes couple resonantly to an acoustic mode via forward Brillouin scattering in BSIT. A strong optical control field near the lower-frequency optical WGM drives a coherent interaction between the acoustic and the higher-frequency optical WGMs, inducing a transparency window for a probe field near the higher-frequency optical WGM. A number of remarkable coherent optical phenomena and potential applications are possible, such as light storage, dark modes and frequency conversions^21^,^22^,^23^. In addition, due to the phase-matching requirement for travelling waves in SBS, the coherent photon–phonon conversion is only allowed for waves propagating in certain directions. We have taken advantage of this property to demonstrate the non-reciprocal storage and retrieval of a coherent optical field. These results indicate that SBS is an excellent candidate for applications in photonic integrated circuits. With the acoustic vibrations cooled to their motional ground states, applications in a quantum regime, such as single-photon storage and frequency conversion, also become possible^24^,^25^,^26^.

## Results

### Stimulated Brillouin scattering

**Figure 1:**
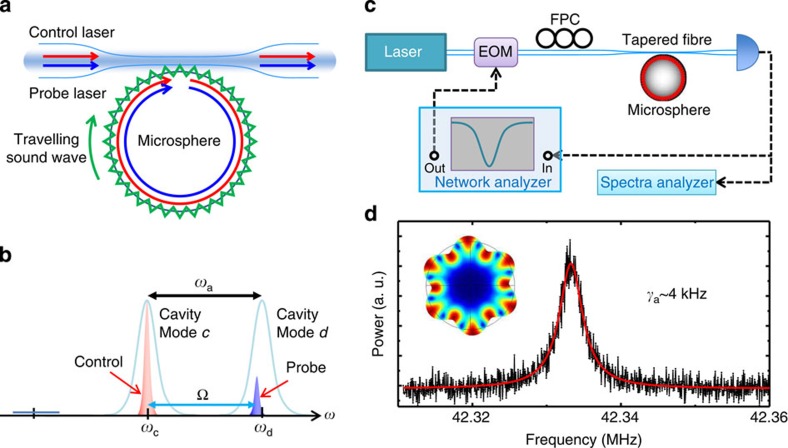
Experimental set-up for Brillouin scattering in a silica microsphere. (**a**) Schematic illustration of the light–acoustic wave interaction in a microsphere. The optical modes of the microsphere are excited by the control and probe lasers through the tapered fibre. These modes interact with the travelling acoustic wave via the forward Brillouin scattering. (**b**) Spectrum diagram of the coherent photon–phonon interaction: the control laser is near resonance with the lower-frequency mode, and the probe field is on resonance with another cavity mode, whereas the Stokes process is suppressed. (**c**) The experimental set-up. EOM, electro-optical modulator; FPC, fibre polarization controller. (**d**) The spectrum of a typical acoustic mode at 42.3 MHz in the microsphere when only the control laser was fixed on resonance with *ω*_*c*_. Inset: simulated deformation of microsphere at the equator for acoustic WGM with *m*_*a*_=6.

In a silica microsphere resonator, there are optical and acoustic WGMs that propagate along the surface (Fig. 1a). Both optical and acoustic WGMs are characterized by the orbital angular momentum mode number, *m*. When the acoustic WGM (*a*) and two optical WGMs (*c* and *d*) satisfy the energy and momentum conservations, such that *ω*_*a*_=*ω*_*d*_−*ω*_*c*_ and *m*_*a*_=*m*_*d*_−*m*_*c*_, photons can be scattered between the two optical resonances through Brillouin scattering^15^. In this work, we focus on the forward SBS, in which *m*_*c*_ and *m*_*d*_ have the same sign and both optical modes are coupled to travelling waves in the same direction through the tapered fibre, as depicted in Fig. 1a,c. As schematically illustrated in Fig. 1b (more detailes in Supplementary Fig. 1 and Supplementary Note 1), the SBS process, for which the control laser pumps on the lower-frequency optical mode, leads to phonon absorption and anti-Stokes photon generation, whereas the Stokes process is inhibited.

Triply resonant SBS was observed for a control laser wavelength near 1,562 nm in a silica microsphere with a radius of 98 μm. We detected the scattered anti-Stokes light by measuring its beating signal with the control laser field. The corresponding power density spectrum was monitored with a spectrum analyser, as shown in Fig. 1d. The Lorentzian lineshape observed indicates an acoustic WGM with a frequency of *ω*_*a*_/2*π*=42.3 MHz and a linewidth of *γ*_*a*_/2*π*=4 kHz (acoustic *Q*_*a*_≈10,600). The acoustic WGM was also further verified by measuring directly the scattered Stokes light (Supplementary Fig. 2). Using the acoustic velocity in silica, we determined the orbital angular momentum mode number of the acoustic WGM to be *m*_*a*_=6 (inset of Fig. 1d).

### Brillouin-scattering-induced transparency

**Figure 2:**
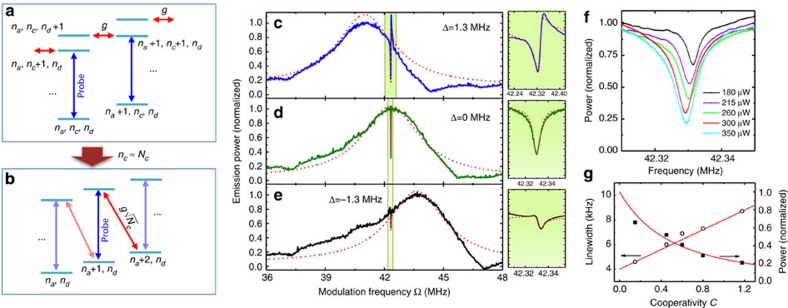
Mechanism and observation of BSIT. (**a**–**b**) Energy diagram for the triply resonant Brillouin scattering discussed in the text. (**c**–**e**) Emission power from the higher-frequency optical mode obtained when we scanned the probe frequency by sweeping the modulation frequency Ω of the control laser for several values of control detuning, Δ=1.3,0,−1.3 MHz. Although the peak of the cavity resonance shifts with Δ, the sharp BSIT dip was always observed at *δ*=0. The incident control power used was 0.3 mW. The insets show the spectral response near *δ*=0 with an expanded frequency scale. The short-dashed lines are the results of calculations using the parameters *k*_*d*_/2*π*=3.5 MHz, *γ*_*a*_/2*π*=0.004 MHz and 
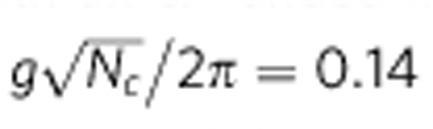
, 0.05, 0.03 MHz, respectively. (**f**) The emission power from the higher-frequency optical mode near *δ*=0 for five different powers of the control beam from 0.18 mW up to 0.35 mW. (**g**) The spectral linewidth and depth of the BSIT dip as a functions of the cooperativity *C*, as derived from **f**. The solid lines in **g** represent the theoretically expected values.

The triply resonant Brillouin scattering discussed above can lead to coherent optical phenomena, such as BSIT, which is an analogue to the well-known electromagnetically induced transparency in atomic system. In a simple and intuitive picture, the control and probe induce a coherent vibration of the acoustic mode. BSIT arises from the destructive interference between the probe field and the anti-Stokes optical field generated by scattering of the control laser from the coherent acoustic vibration. For our system, this destructive interference prevents the excitation of the higher-frequency optical mode. For a detailed theoretical description of BSIT, we consider the system Hamiltonian including the non-linear photon–phonon interaction


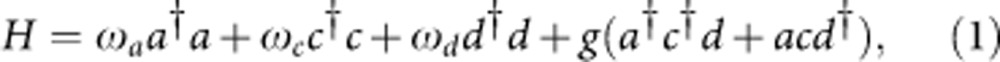


where *a* is Boson operator of the acoustic mode and *c*,*d* are Boson operators of the optical modes (see Fig. 1b), *g* is the single-photon Brillouin coupling rate, which is non-zero only when the phase-matching condition is satisfied. The energy diagram of the system is schematically illustrated in Fig. 2a, where the energy levels are described by phonon and photon Fock states |*n*_*a*_, *n*_*c*_, *n*_*d*_›, where *n*_*a*(*c*,*d*)_ is the number of phonons (photons) and Brillouin scattering induces transitions between |*n*_*a*_, *n*_*c*_, *n*_*d*_ +1› and |*n*_*a*_+1, *n*_*c*_+1, *n*_*d*_›. For a strong control laser such as 
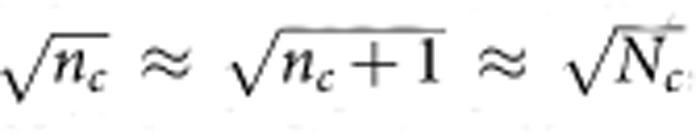
, the energy diagram can be simplified as Fig. 2b, which has resemblance to the Λ-type system in atomic electromagnetically induced transparency.

Under the strong control laser driving, the Hamiltonian is effectively simplified to (see Supplementary Note 2)


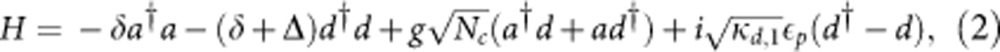


where the acoustic mode and the higher-frequency optical mode (mode *d*) are coupled linearly, with the effective coupling rate determined by the photon number of lower-frequency optical mode. Here, *ε_p_* denotes the weak probe field with a frequency of *ω*_*p*_ coupling to mode *d* (see Fig. 1b), Δ=*ω*_*a*_+*ω*_*l*_−*ω*_*d*_ and *δ*=*ω*_*p*_−*ω*_*l*_−*ω*_*a*_ are relevant frequency detunings, where *ω*_*l*_ is the frequency of the control laser. The steady-state intracavity power spectrum is given by,


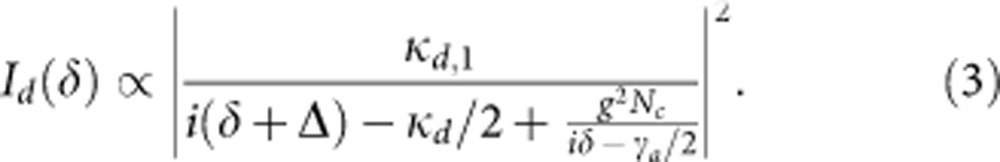


The intracavity power of mode *d* is modified by the coherent photon–phonon interaction, giving rise to significant changes in the intracavity power when 
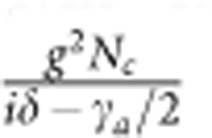
 is comparable to *κ*_*d*_. It is convenient to introduce the cooperativity defined as 
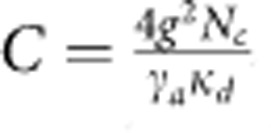
, a dimensionless parameter that characterizes the relative strength of the coherent photon–phonon interaction. *C* also characterizes quantitatively the BSIT process.

To observe BSIT in our system, we probed the intracavity power spectrum in the higher-frequency optical mode using a heterodyne detection technique, with the control laser serving as the local oscillator. We generated the probe field by phase modulating the control laser with an EOM, with modulation frequency Ω. Using a network analyser, the intracavity power can be extracted from the heterodyne signal with modulation frequency Ω. As shown in Fig. 2c–e, we investigated the dependence of the BSIT response on detuning Δ by adjusting *ω*_*l*_ with a fixed control power of *P*=300 μW. The transparency window was observed when the two-photon detuning is near the mechanical resonance frequency, *ω*_*p*_−*ω*_*l*_≈*ω*_*a*_. The lineshapes of the BSIT response, as shown in the expanded spectra in Fig. 2c–e, demonstrate directly the optical interference between the intracavity probe field and the anti-Stokes field. A theoretical calculation of the BSIT response based on equation (3) leads to estimated values of *C*=5.6, 0.71 and 0.26 for Δ=1.3, 0 and −1.3 MHz, respectively. The triply resonant Brillouin Scattering system enables us to achieve a cooperativity near 6 with a control laser power about 300 μW. Much greater control powers are needed for the achievement of similar cooperativity in WGM-type optomechanical resonantors that do not satisfy the triple resonant conditions.

The strongest cooperativity was not observed at Δ=0, because the triply resonant condition was not exactly satisfied in the experiment, as we estimated *ω*_*d*_−*ω*_*c*_−*ω*_*a*_≈1.3 MHz. Further studies of the BSIT dip at Δ=0 are presented in Fig. 2f, for which the control laser power was varied from 180 to 350 μW. BSIT dips of increasing depths and widths were observed, which can be fitted numerically with a simple Lorentzian lineshape. As shown in equation (3), the linewidth of the BSIT dip is given by (1+*C*)*γ*_*a*_ and the relative depth of the BSIT dip is given by 
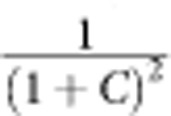
. However, we found that *C* derived from the numerical fits was not proportional to the input control power because of the thermal effect, which slightly changes the triply resonant condition.

### Non-reciprocal light storage

Owing to the coherent Brillouin interaction, the coherent conversion between photons and acoustic phonons can be used for light storage^23^. In contrast to the previously studied optomechanics driven by radiation pressure and gradient forces, in which the mechanical vibrations can couple to a variety of optical modes, Brillouin scattering is possible only for specific modes that satisfy the energy and momentum conservation conditions. The input control laser coupling to clockwise (CW) optical mode only permits interactions between CW acoustic phonons and photons, whereas the counter-clockwise (CCW) probe field or acoustic wave is decoupled from the control field. Therefore, the requirement of phase matching for the Brillouin scattering process gives rise to the breaking of the relevant reversal symmetry, leading to non-reciprocal non-linear optical processes that are switchable by the control laser.

**Figure 3:**
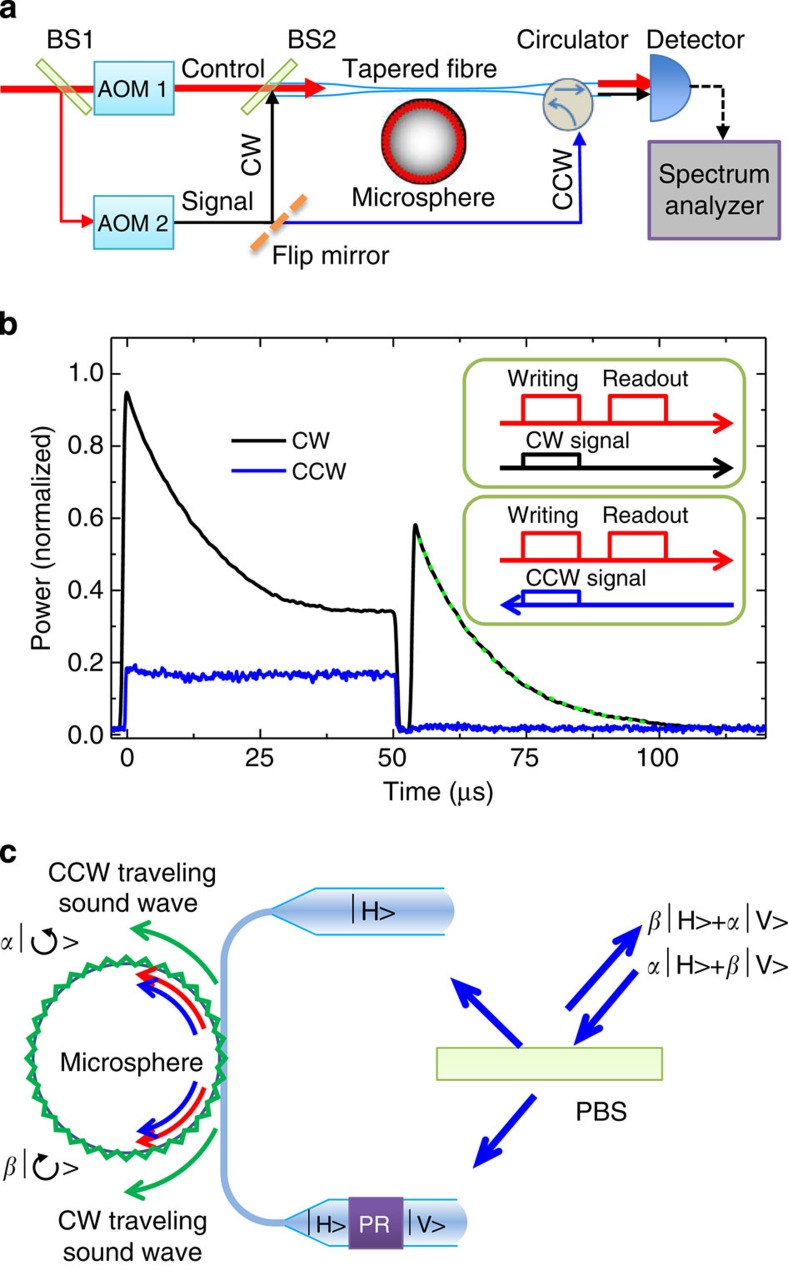
Non-reciprocal light storage and proposed quantum memory design. (**a**) The experimental set-up for the non-reciprocal light storage. The CW signal is combined with the CW control laser through a beam splitter (BS2). The CCW signal is launched into the fibre through a circulator. (**b**) The measured intracavity signal power during the storage and retrieval processes. The black and blue lines correspond to the input signal with different input directions. Inset: the pulse sequences for writing, readout and signal, with the arrows indicating the propagation direction. (**c**) Schematic diagram of single-bit quantum memory based on circulating acoustic phonons. PBS, polarization beam splitter; PR, polarization rotator.

To demonstrate this non-reciprocity, we studied the light storage of CW or CCW signal fields with a fixed CW control laser. As shown in Fig. 3a, the control and signal fields are switched and frequency shifted by two acousto-optic modulators (AOM 1 and 2), with frequency difference matching *ω*_*a*_. The control laser pulses, including writing and readout, are coupled to the CW mode only. The signal pulse is coupled to either CW or CCW mode by moving a flip mirror. The sequences of writing, readout and signal pulses are shown in the inset of Fig. 3b. When the triply resonant condition is satisfied, the CW signal pulse will be converted to a CW acoustic wave coherently via the beam-splitter-type Brillouin coupling shown in equation (2). The coherence nature of the interconversion between optical and mechanical excitations have been demonstrated in an earlier study of mechanical breathing modes^21^,^27^. As shown in Fig. 3b, the CW signal (black line) was stored during the writing pulse and retrieved during the readout pulse after a delay of 3 μs, which is much longer than the cavity photon lifetime. The exponential decay of the signal intensity during the writing pulse results from the underlying dynamical BSIT process. A similar phenomenon has also been reported for mechanical breathing modes in a silica microsphere in ref. 28. The decay time of the converted signal is 14 μs, as determined from an exponential fit (green dashed line in Fig. 3b). In addition, the bandwidth for light storage is determined by the transparency bandwidth of BSIT, (1+*C*)*γ*_*a*_, where *C* is proportional to the input control power (see Fig. 2f).

In comparison, for the CCW-launched signal (blue line), there was no light retrieval during the readout pulse. It should be noted that the flat background signal during the writing pulse originated from the beating between the writing field and the reflected signal field from the end facet of the circulator.

## Discussion

In the previous section, we provided experimental evidences of coherent and non-reciprocal photon–phonon interconversion arising from Brillouin scattering in a silica microsphere, though the experiments at room temperature are affected by the thermal acoustic phonons. Since the interaction between the signal field and the acoustic wave with a strong control field is in the form of 
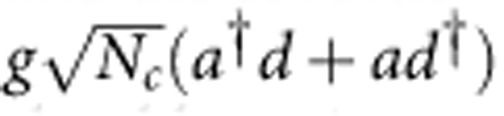
 (equation (2)), the coherent interconversion should persist even when the signal field is at the single-photon level^25^. In particular, single-photon storage becomes possible when the relevant acoustic modes are cooled down to their ground states. For *ω*_*a*_/2*π*=1 GHz and at *T*=0.5 K, the thermal phonon occupation is near 10. In this case, the ground state cooling requires a cooperativity *C* >10, which is achievable for silica microspheres, given the reduced thermal bistability and improved mechanical *Q* factor at the low temperature. Note that the state transfer between a motional state and a microwave field in the quantum regime has already been demonstrated recently in an electromechanical system^24^.

Because of the momentum conservation required for SBS, the degenerate CW and CCW acoustic modes can be written and readout independently by the corresponding CW and CCW control laser. Assuming degenerate CW and CCW optical WGMs in a high-quality whispering gallery microresonator, we can treat a pair of WGMs as one bit of binary information carriers. Here we also proposed a design of quantum memory for polarization-encoded photon state. As illustrated in Fig. 3c, with the use of the polarization beam splitter and the polarization rotator, horizontally and vertically polarized input photon states can individually couple to CW and CCW optical modes. These states can be stored as the CW and CCW acoustic waves and be readout, separately. In principle, the input state, a superposition of polarization state *α*|*H*›+*β*|*V*›, can be stored as a superposition of circulating acoustic state *α*|›+*β*|›. In the reversal of the storage process, the states can be readout by being converted to the photon state *α*|*V*›+*β*|*H*› after tens of microseconds.

Brillouin scattering in whispering-gallery microresonators is promising in several aspects. (a) Brillouin scattering enables the optical coupling to acoustic waves with frequency ranging from a few MHz to 11 GHz, thereby providing a diverse platform for coherent light-matter interactions. (b) The triply resonant configuration can greatly enhance the Brillouin scattering, thereby reducing the power consumptions^15^,^17^. (c) Phase matching for the travelling waves enables non-reciprocal optical processes, thus offering potential application in an all-optical integrated isolator and circulator devices^29^,^30^. Our studies pave the way towards the coherent coupling between photons and acoustic phonons and should stimulate further investigations of non-reciprocity and memory at the quantum level.

During the preparation of this manuscript, a similar work has been reported on the arXiv^31^.

## Methods

### Experiment method

The experimental set-up is schematically illustrated in Fig. 1c. All experiments were performed at room temperature and at atmospheric pressure. Silica microspheres were fabricated by melting a tapered fibre with a CO_2_ laser. Optical WGMs in the microsphere were excited through the evanescent field of a tapered optical fibre with a tunable narrow-linewidth (<300 kHz) external-cavity laser at the 1,550-nm band. The coupling strength between the tapered fibre and the optical WGMs can be adjusted by changing the air gap between the fibre and microsphere, which was controlled by a high-resolution translation stage. The optical output was detected using a low-noise photoreceiver, connected to a digital oscilloscope for the measurement of the transmission spectra or to a spectrum analyser for the identification and characterization of the acoustic modes. For the BSIT experiment, an acoustic mode with *ω*_*a*_/2*π*=42.3 MHz and *γ*_*a*_/2*π*=4 kHz (acoustic *Q*_*a*_≈10,600) was used. The probe field was generated by modulating the control laser through an EOM. A network analyser provided the modulation signal for the EOM and also measured the power spectral density of the beating signal between the control and probe field.

The experiment on optical non-reciprocity was carried out in another silica microsphere with *ω*_*a*_/2*π*=152.7 MHz and *γ*_*a*_/2*π*=15 kHz (acoustic *Q*_*a*_≈10,180). As shown in Fig. 3a, the control and probe field are generated by two separate acousto-optic modulators (AOM 1 and 2). The writing and readout control pulse sequence was generated by AOM 1, in which a laser beam was frequency shifted by −80 MHz. The signal pulse, which was in synchronization with the writing pulse, was obtained with the use of AOM 2, which frequency shifted the laser beam +72.7 MHz. The peak powers of the writing and readout pulses were both 1 mW, and the peak power of the signal pulse was 20 nW. The durations of writing and reading pulse were 50 and 80 μs, respectively. The two pulses were separated by 3 μs. The forward and backward going signal pulses were separately coupled into the fibre. The power of the optical output was measured with a spectrum analyser operating in a gated-detection mode. The resolution bandwidth was set to 10 MHz.

## Author contributions

C.-H.D. and Z.S. contribute equally to this work. C.-H.D. and Z.S. prepared the microsphere and carried out the experiment measurements. C.-L.Z., Y.-L.Z. and W.F. provided the theoretical support and analysis. C.-L.Z. and C.-H.D. wrote the manuscript. C.-H.D, C.-L.Z. and G.-C.G. supervised the project. All authors contributed extensively to the work presented in this paper.

## Additional information

**How to cite this article:** Dong, C.-H. *et al*. Brillouin-scattering-induced transparency and non-reciprocal light storage. *Nat. Commun.* 6:6193 doi: 10.1038/ncomms7193 (2015).

## Supplementary Material

Supplementary Information Supplementary Figures 1-3, Supplementary Table 1, Supplementary Notes 1-4
