# Supplementary material for: Brillouin-scattering-induced transparency and non-reciprocal light storage
Source: Nat Commun. 2015 Feb 4;6:6193. doi: 10.1038/ncomms7193 (PMC4327558; doi:10.1038/ncomms7193)
Supplement: Supplementary Information — Supplementary Figures 1-3, Supplementary Table 1, Supplementary Notes 1-4 [file ncomms7193-s1.pdf]

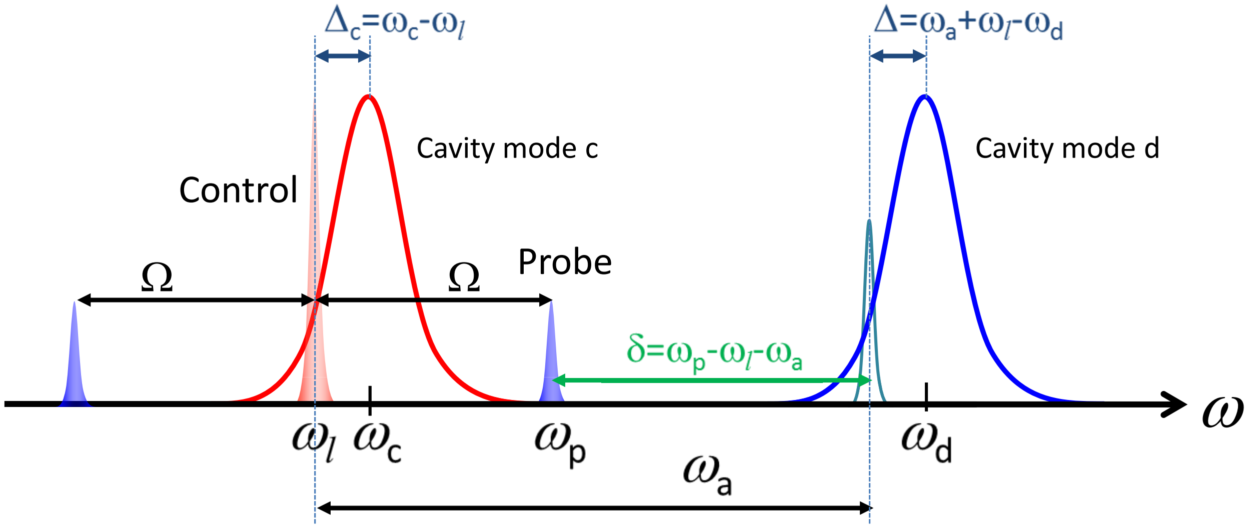

Supplementary Figure 1: Frequency relationship of the triple resonant system for Brillouin-scattering-induced transparency.

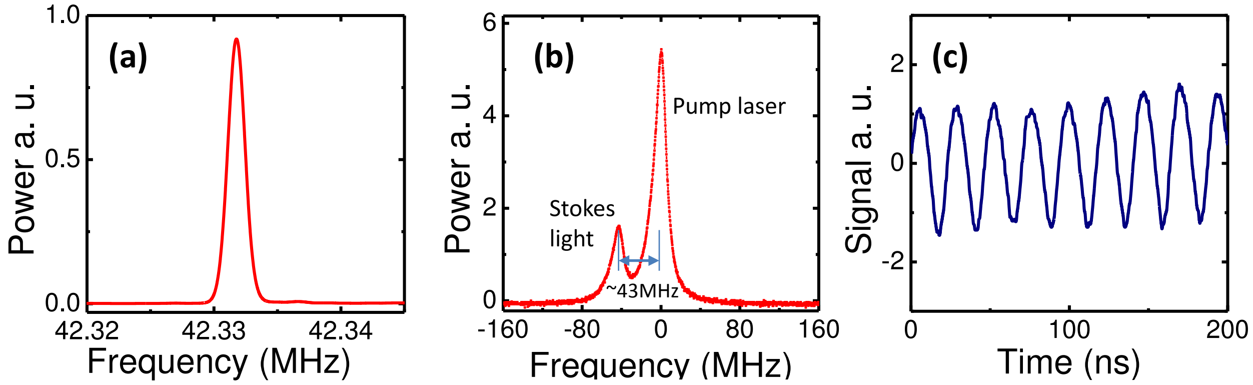

Supplementary Figure 2: **Optical and electrical spectra for Brillouin scattering by a 42.3 MHz acoustic wave.** (a) The spectrum of the typical acoustic mode when the control laser pumped at the higher-frequency optical mode. (b) The optical spectrum measured by Fabry-Perot spectrum analyzer. (c) The time-dependent detected optical power represents the beating signal between the two optical signals. These results are obtained in a sphere of diameter 196 μm with a control wavelength around 1562 nm.

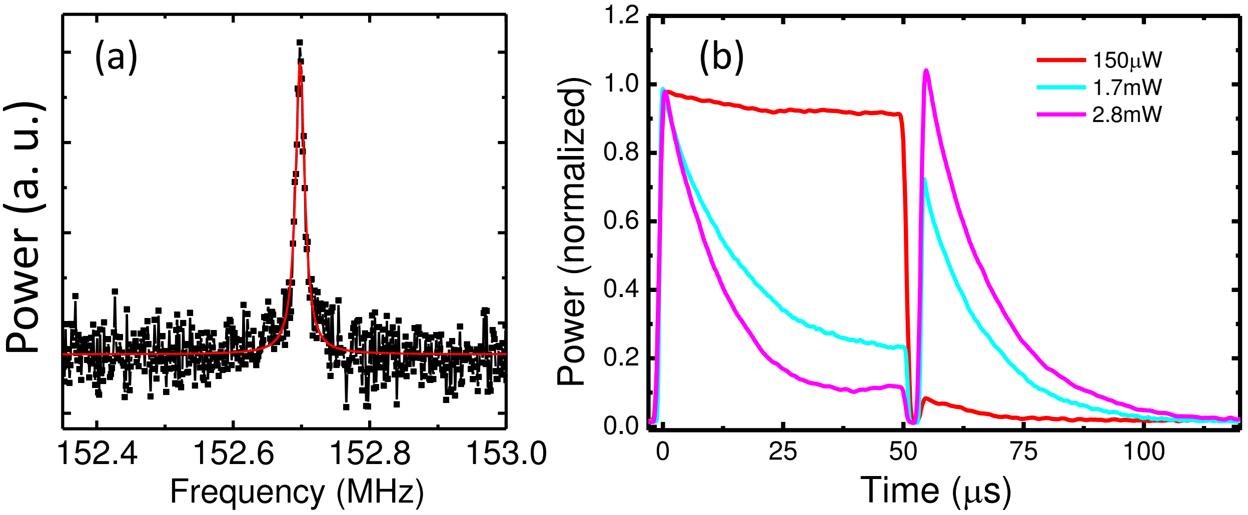

Supplementary Figure 3: **Non-reciprocal light storage experiment.** (a) The spectra of the acoustic mode used for the light storage experiment. The high-Q acoustic mode has frequency of $\omega_a/2\pi = 152.7$ MHz and linewidth of $\gamma_a/2\pi = 15$ kHz, corresponding to an acoustic Q-factor $Q_a \approx 10180$. (b) The storage and retrieval of signal light with various pump power.

Supplementary Table 1: The symbols of frequencies in the triple resonances system.

Symbol	Expression	Description
ω_a	/	frequency of acoustic mode a
ω_c	/	frequency of optical mode c
ω_d	/	frequency of optical mode d
ω_l	/	control laser frequency
Ω	/	EOM modulation frequency
ω_p	$\omega_l + \Omega$	probe laser frequency
Δ_c	$\omega_c - \omega_l$	detuning between control laser and optical mode c
Δ	$\omega_a + \omega_l - \omega_d$	detuning between the expected transparency and optical mode d
δ	$\omega_p - \omega_l - \omega_a$	detuning between probe laser and anti-Stokes light of control laser

Supplementary Note 1. The Brillouin scattering system

As described in the main context, the system consists of: (1) acoustic wave a, (2) two optical cavity modes c and d. There are two lasers input to the system, the control laser near resonant to cavity mode c and the probe laser near resonant to cavity mode d. The whole system is inevitably complicated, thus we plotted the detailed illustration of the modes and lasers in Supplementary Fig. 1. The explanation to the symbols and expressions is provided in Supplementary Table 1.

In Supplementary Fig. 1, the two cavity modes are shown by Red and Blue Lorentz peaks. The control laser is denoted by a narrow pink peak, with the frequency of ω_l. The probe laser is generated by EOM (modulation frequency Ω) sideband of the control laser, as shown by the blue tiny peaks around the control laser.

The triple resonant condition is satisfied when $\omega_d - \omega_c = \omega_a$, corresponding to Δ_c and Δ both equal to 0.

The Brillouin scattering induced transparency happens when $\omega_p - \omega_l = \omega_a$, corresponding to $\delta = 0$.

Supplementary Note 2. Theoretical Derivation

Coupled-oscillator equations for Microsphere-microfiber

For the microsphere microcavity coupling to taper fiber, the interaction can be described by

$$H = \omega_c c^\dagger c + \omega_d d^\dagger d + i\sqrt{\kappa_{c,1}}\epsilon_l (c^\dagger e^{-i\omega_l t} - c e^{i\omega_l t}) + i\sqrt{\kappa_{d,1}}\epsilon_p (d^\dagger e^{-i\omega_p t} - d e^{i\omega_p t}). \quad (1)$$

Here, the frequencies have been explained in the main context and first section in the Supplementary Materials. The operators $c\,(c^\dagger)$ and $d\,(d^\dagger)$ are the annihilation (creation) operators of the optical cavity, which have been explained in the main context. The parameter $\kappa_{c,1}$ ($\kappa_{d,1}$) is the coupling strength between the cavity mode and waveguide, which depends on the overlap of electromagnetic field between cavity and waveguide mode fields. Usually, the spatial distributions of optical modes c and d are different, thus the coupling strengths are different.

For the system without Brillouin scattering interaction, the control and probe cavity

fields can be solved as

$$\frac{d}{dt}c = (-i\omega_c - \kappa_c/2)c + \sqrt{\kappa_{c,1}}\epsilon_l e^{-i\omega_l t}, \tag{2}$$

$$\frac{d}{dt}d = (-i\omega_d - \kappa_d/2)d + \sqrt{\kappa_{d,1}}\epsilon_l e^{-i\omega_p t}. \tag{3}$$

The transmissions of the laser are

$$E^c_{out} = \epsilon_l - \sqrt{\kappa_{c,1}}c, \tag{4}$$

$$E^d_{out} = \epsilon_p - \sqrt{\kappa_{d,1}}d. \tag{5}$$

Linearized Hamiltonian

When the phase matching condition is satisfied, the forward SBS leads to coherent interaction between the optical and acoustic modes. The Hamiltonian of the system with control and probe light input is

$$\begin{aligned} H =& \omega_a a^\dagger a + \omega_c c^\dagger c + \omega_d d^\dagger d + g(a^\dagger c^\dagger d + acd^\dagger) \\ &+ i\sqrt{\kappa_{c,1}}\epsilon_l(c^\dagger e^{-i\omega_l t} - ce^{i\omega_l t}) + i\sqrt{\kappa_{d,1}}\epsilon_p(d^\dagger e^{-i\omega_p t} - de^{i\omega_p t}). \end{aligned} \tag{6}$$

Here, the a and $a^\dagger$ denote the operators of acoustic wave.

For very strong control laser pumping to mode c, under the condition that the probe laser power and the amplitude of acoustic wave are very weak, we can treat the control mode field classically as a complex number. Under the non-depletion approximation of the nonlinear three-wave mixing, the dynamics of control mode c can be described by Eq. 2, where the mode is not influenced by SBS. In our experiment, the duration of control pulse $\tau_{pump} \gg 1/\kappa_c$, thus we have the steady state solution of mode c as

$$\langle c \rangle = \sqrt{N_c}e^{-i\omega_l t} = \left| \frac{\sqrt{\kappa_{c,1}}\epsilon_l}{-i\Delta_c - \kappa_c/2} \right| e^{-i\omega_l t}, \tag{7}$$

where the detuning $\Delta_c = \omega_c - \omega_l$ [see also Supplementary Fig. 1 and Supplementary Table 1].

By substituting this complex number into the Hamiltonian, we obtain the linearized photon-phonon interaction as

$$H = \omega_a a^\dagger a + \omega_d d^\dagger d + g\sqrt{N_c}(ad^\dagger e^{-i\omega_l t} + a^\dagger de^{i\omega_l t}) + i\sqrt{\kappa_{d,1}}\epsilon_p(d^\dagger e^{-i\omega_p t} - de^{i\omega_p t}). \tag{8}$$

In the rotating frame of $H_0 = \omega_p d^\dagger d + (\omega_p - \omega_l) a^\dagger a$, we have

$$H_{eff} = -\delta a^\dagger a - (\delta + \Delta) d^\dagger d + g\sqrt{N_c}(a^\dagger d + a d^\dagger) + i\sqrt{\kappa_{d,1}}\epsilon_p (d^\dagger - d), \tag{9}$$

where the detuning are $\delta = \omega_p - \omega_l - \omega_a$ and $\Delta = \omega_a + \omega_l - \omega_d$ [see also Supplementary Fig. 1 and Supplementary Table 1].

Brillouin-scattering-induced Transparency

As studies above, under the strong control laser field, the optical mode d and acoustic mode a are coupled linearly. Therefore, coherent conversion between the photon and phonon can be realized. The dynamics of the probe light mode and acoustic mode are

$$\frac{d}{dt} a = (i\delta - \gamma_a/2) a - ig\sqrt{N_c} d, \tag{10}$$

$$\frac{d}{dt} d = [i(\delta + \Delta) - \kappa_d/2] d - ig\sqrt{N_c} a + \sqrt{\kappa_{d,1}}\epsilon_p. \tag{11}$$

In the steady state $\frac{d}{dt}a = \frac{d}{dt}d = 0$, we have

$$a = \frac{ig\sqrt{N_c}}{i\delta - \gamma_a/2} d, \tag{12}$$

$$d = \frac{-\sqrt{\kappa_{d,1}}\epsilon_p}{i(\delta + \Delta) - \kappa_d/2 + \frac{g^2 N_c}{i\delta - \gamma_a/2}}. \tag{13}$$

From the experiments, the measured heterodyne signal is corresponding to the intensity of intracavity field

$$I_d(\delta) \propto \left| \frac{\kappa_{d,1}}{i(\delta + \Delta) - \kappa_d/2 + \frac{g^2 N_c}{i\delta - \gamma_a/2}} \right|^2. \tag{14}$$

Supplementary Note 3. Verification of Brillouin scattering

When the two optical modes (c and d) and the acoustic mode satisfy the triply resonant condition, there are actually two different processes of the Brillouin scattering:

(1) The control laser is coupled to lower-frequency mode c: the anti-Stokes scattered light is coupled to mode d and accompanied with absorption of acoustic wave. This process cools the amplitude of acoustic wave.

(2) The control laser is coupled to higher-frequency mode d: the Stokes scattering process leads to the generation of phonon-photon pair, corresponding to the parametric down conversion. This process amplifies (heats) the amplitude of acoustic wave.

In the main context, we only concern about the first case. Figure 1(d) shows the spectra obtained from spectrum analyzer for the control laser fixed on-resonance with ω_c. The peak was originated from the beating between control and scattered anti-Stokes lights. Due to low thermal excitation of acoustic phonon, the anti-Stokes signal is very weak compared with the control field.

To confirm the observation of the Brillouin scattering, we also studied the second case. Since the acoustic wave is amplified, the Stokes signal in second case is much stronger than the anti-Stokes signal in first case, as shown in Supplementary Fig. 2(a). To demonstrate the Brillouin scattering induced Stokes lights directly, we measured the optical spectrum by a tunable Fabry-Perot spectrum analyzer (Thorlabs SA200-14A). Shown in Supplementary Fig. 2(b) is the spectrum of cavity emission with strong control laser driving on-resonance with ω_d. There is a prominent peak on the red side of the control light, indicating the Stokes scattering light. The frequency difference between the peaks is about 43 MHz, which agrees well with the results by beating signal as shown by Supplementary Fig. 2(c). Additionally, the absence of the anti-Stokes sideband in the optical spectrum as well as the absence of higher harmonics clearly confirms the triply resonant condition [Fig. 1(b) in the main context].

Supplementary Note 4. Non-reciprocal light storage

In the main context, the light storage and readout are studied for CW and CCW signal. The results shows the signal retrial after 3 μs storage in the microsphere. These results demonstrated the non-reciprocal light storage to the acoustic wave. Here, more experimental data are provided in Supplementary Fig. 3(a-b) to support the non-reciprocal light storage. Shown by different curves in Supplementary Fig. 3(b) are the light storage and retrieval for various control laser power. The similar input signal powers were used for the experiments presented in the main text. From the results, we can find that the higher control laser power corresponding to the stronger decline and greater retrieval signal. These can be explained that the cooperativity of interaction between probe light and acoustic wave is proportional to the control laser power. These results are consistent with the results of Fig. 2(f) in the main context.
